# Moral frameworks of commercial surrogacy within the US, India and Russia

**DOI:** 10.1080/26410397.2021.1878674

**Published:** 2021-03-01

**Authors:** Marcin Smietana, Sharmila Rudrappa, Christina Weis

**Affiliations:** a Research Associate, ReproSoc, Cambridge University, Cambridge, UK. *Correspondence:* ms935@cam.ac.uk; b Professor, Department of Sociology, University of Texas, Austin, USA; c Research Fellow, Centre for Reproduction Research, De Montfort University, Leicester, UK

**Keywords:** surrogacy, surrogate mothers, moral frameworks, repronational histories, clinical labour, reproductive markets, USA, India, Russia

## Abstract

In this paper, we draw on three ethnographic studies of surrogacy we carried out separately in different contexts: the western US state of California, the south Indian state of Karnataka, and the western Russian metropolis of St Petersburg. In our interviews with surrogate mothers, intended parents, and surrogacy professionals, we traced the meanings and ideologies through which they understood the clinical labour of surrogacy. We found that in the US, interviewed surrogates, intended parents and professionals understood surrogacy as an exchange of both gifts and commodities, where gift-giving, reciprocity, and relatedness between surrogates and intended parents were the major tropes. In India, differing narratives of surrogacy were offered by its different parties: whilst professionals and intended parents framed it as a win-win exchange with an emphasis on the economic side, the interviewed surrogate mothers talked about surrogacy as creative labour of giving life. In Russia, approaches to surrogacy among the interviewed surrogate mothers, professionals and intended parents overlapped in framing it as work and a businesslike commodity exchange. We suggest these three different ways of ethical reasoning about the clinical labour of surrogacy, including justifications of women’s incorporation into this labour, were situated in local moral frameworks. We name them “repro-regional moral frameworks”, inspired by earlier work on moral frameworks as well as on reproductive nationalisms and transnational reproduction. Building on these findings, we argue that any international or global regulation of surrogacy, or indeed any moral stance on it, needs to take these local differences into account.

## Résumé

Dans cet article, nous nous inspirons de trois études ethnographiques sur la maternité de substitution que nous avons réalisées séparément dans différents contextes: l’État de Californie à l’ouest des États-Unis, l’État de Karnataka au sud de l’Inde et la métropole de Saint-Pétersbourg en Russie occidentale. Dans nos entretiens avec les mères porteuses, les futurs parents et les professionnels de la maternité de substitution, nous avons recherché les significations et les idéologies dont ils se servaient pour comprendre le travail clinique de la maternité de substitution. Nous avons constaté qu’aux États-Unis, les mères porteuses, les futurs parents et les professionnels interrogés comprenaient la maternité de substitution comme un échange à la fois de dons et de produits, où le don, la réciprocité et le lien entre les mères porteuses et les futurs parents étaient les principaux thèmes. En Inde, les diverses parties ont proposé différentes descriptions de la maternité de substitution: si les professionnels et les futurs parents la concevaient comme un échange où chacun y trouve son compte en mettant l’accent sur son côté économique, les mères porteuses interrogées ont parlé de la maternité de substitution comme d’un travail créatif pour donner la vie. En Russie, les mères porteuses, les professionnels et les futurs parents avaient des conceptions de la maternité de substitution qui se recoupaient et ils la considéraient comme une activité économique et un échange commercial de produits. Nous suggérons que ces trois différents raisonnements éthiques sur le travail clinique de la maternité de substitution, y compris les justifications de l’incorporation de ces femmes dans ce travail, s’inscrivaient dans des cadres moraux locaux. Nous les appelons des cadres moraux repro-régionaux, inspirés par un travail antérieur sur les cadres moraux ainsi que sur les nationalismes reproductifs et la reproduction transnationale. Nous basant sur ces conclusions, nous avançons que toute réglementation mondiale ou internationale de la maternité de substitution, ou même toute position morale à son propos, doit prendre en compte ces différences locales.

## Resumen

En este artículo, nos basamos en tres estudios etnográficos de subrogación que realizamos por separado en diferentes contextos: el estado occidental de California en EE. UU., el estado de Karnataka en India meridional, y la metrópolis occidental de San Petersburgo en Rusia. En nuestras entrevistas con madres subrogadas, padres futuros y profesionales de subrogación, encontramos los significados y las ideologías por medio de los cuales entendieron la labor clínica de gestación subrogada. Hallamos que en Estados Unidos, las madres subrogadas, padres futuros y profesionales entrevistados entendían la subrogación como el intercambio de regalos e insumos, donde dar regalos, reciprocidad y parentesco entre madres subrogadas y padres futuros eran los principales tropos. En India, las diferentes partes ofrecieron diferentes narrativas de subrogación: mientras que los profesionales y padres futuros la definen como un intercambio donde se benefician todas las partes, con énfasis en el aspecto económico, las madres subrogadas entrevistadas hablaron sobre subrogación como la labor creativa de dar vida. En Rusia, los enfoques de las madres subrogadas, profesionales y padres futuros entrevistados con relación a la subrogación coincidieron en definirla como trabajo y como intercambio profesional de insumos. Sugerimos que estas tres formas diferentes de razonamiento ético sobre la labor clínica de subrogación, incluidas las justificaciones de la incorporación de las mujeres en esta labor, estaban situadas en marcos morales locales. Los denominamos *marcos morales repro-regionales*, inspirados por trabajo anterior en marcos morales, así como en nacionalismos reproductivos y reproducción transnacional. A raíz de estos hallazgos, argumentamos que toda regulación internacional o mundial de subrogación, incluso toda postura moral al respecto, debe tomar en cuenta estas diferencias locales.

## Introduction

Commercial gestational surrogacy entails contractual arrangements between women and intended parents, often strangers to each other, where the former serve as surrogate mothers* to carry babies to term. They receive money for their considerable efforts and the latter receive a baby in return. Emerging from a plethora of arrangements, wherein intended parents’ sperm or ova are used, or such gametes are purchased from sex cell banks to which they had been provided by egg or sperm donors, the infants are usually not genetically descended from the surrogate mothers who gestate them. Today commercial surrogacy is a multi-million dollar industry in various countries across the world, raising bioethical concerns regarding designer babies and exploitation of women, and counter-arguments on how these sorts of markets extend the reproductive rights of all actors involved. Importantly though, surrogacy emerges through a new kind of labour, which has been theorised as *clinical labour*,^1,2^ alongside other new kinds of work such as that carried out by gamete donors, organ donors, or subjects of medical trials. Such labour involves being a subject of in-vivo extractive processes, where surrogate mothers’ biological processes of oogenesis and gestation are managed for the clients’ benefit. Carrying out the clinical labour^1^ of surrogacy may impact directly on the sexual and reproductive health of the surrogate mothers and egg donors involved, as much as it can also shape the definitions of intended parents’ reproductive wellbeing. However, relationships between reproductive workers and intended parents often develop in unequal contexts of stratified reproduction,^3^ where reproductive labourers incorporate themselves into surrogacy markets both under the pressures of the local (re-)productive economies ^4–8^ and through their own ethical reasoning that they negotiate in locally situated and socially acceptable ways.^9–13^

In this comparative paper, we look at three different contexts of surrogacy within the US, India and Russia. Whilst a common denominator across the three locations we studied was that surrogate mothers received financial compensation for their labour, we trace how the involved actors in each context interpreted the reproductive market arrangements in different ways, and how they justified their participation in those local markets of clinical labour. Thus, we look at how local moral frameworks shaped how surrogate mothers understood their work as surrogate mothers, as well as how other participants in the surrogacy process such as intended parents and surrogacy professionals understood that work. We trace meanings and indeed also broader ideologies through which our interviewees understood surrogacy, while a comparison of the specific conditions of labour in each of the three cases we analyse remains beyond the scope of this paper. We compare how the specific meanings and ideologies built around the clinical labour of surrogacy varied within the three contexts, as well as how this in turn determined the participants’ reasoning throughout their participation in this labour.

The questions we ask appear to be of prime importance as feminist scholars of reproduction and reproductive justice activists have been discussing the possibilities for ethical surrogacy, particularly in the context of transnational surrogacy and global inequalities, ^14,15^ or indeed the possibility of a global regulation of surrogacy, which has been considered not least since the International Forum on Intercountry Adoption and Global Surrogacy in the Hague in 2014. ^8,16–19^ We suggest that any international regulation of surrogacy needs to take into account its local moral frameworks in order to be effective.

In the initial part of the paper, we first discuss the key concepts and literature we are in conversation with through our research. We then introduce the three contexts in which our research took place, followed by methodological details of our three studies. In the findings section, we present our ethnographic accounts of the ethical reasoning undertaken by surrogate mothers, intended parents and surrogacy professionals in the three sites we studied: the western US state of California, the south Indian state of Karnataka, and the western Russian metropolis of St Petersburg. We close by a final discussion section, where we also return to the potential policy implications from our research.

## Key concepts and literature

We build on the concepts of “clinical labour”^1^ and “moral frameworks”,^12^ to foreground interconnections between surrogacy labour markets and individuals’ ethical reasoning. We then link these concepts to work on “repronational histories”^20^ and “transnational reproduction”,^6,21,22^ both of which our research bridges through showing what we call “repro-regional moral frameworks”. We argue that surrogacy participants use local or “repro-regional” moral frameworks in their ethical reasoning about their participation in surrogacy, which in turn has important implications for scholars, activists and policy-makers considering if and how surrogacy should be regulated, not least at the global level.

### Clinical labour

One particularly salient aspect of economic relationships between reproductive workers such as surrogate mothers, on the one hand, and intended parents, on the other, is labour. Framing surrogacy as labour can itself be viewed as part of a moral framework, as it may already position surrogacy as ethically acceptable in some contexts.^4,7,23^ Making the gendered reproductive labour visible has also been proposed by many feminist scholars of reproduction, through examples such as surrogacy^10^ and egg donation.^24^ Such claims have made some consider what sort of labour surrogacy is, and whether it is somehow different from other forms of labour.

Reproductive labour, particularly childcare, has been theorised as a form of paid and unpaid labour performed mostly by women, and in the global North often by migrant women of color.^3,25,26^ In particular, scholars such as Evelyn Nakano Glenn^27^ have shown how racialised histories of settler colonialism continue to determine how reproductive labour is carried out by women of color and how it is devalued.

Reproductive labour can be broadly understood as the plethora of emotional, physical, and psychic life-sustaining work involved in tending to the emotional, intimate, and bodily needs of dependent others.^28–30^ Class-privileged individuals who are unable to birth genetically descended children because of medically or socially indicated factors recruit, through a network of agencies that crisscross the world, working and middle-class women to gestate and birth babies for them. Surrogacy is characterised by stratified reproduction and often follows global neo-colonial routes.^11,31^^,32^^,^^6,7,33^ In the US, though most surrogate mothers and intended parents share white Protestant backgrounds, surrogates tend to be of lower middle-class origins whereas intended parents often come from upper middle classes.^34^ Focusing particularly on how women in India are assimilated into the reproductive economy, Amrita Pande^11^ used the term “mother workers” to describe surrogate mothers. Like factory workers, mother workers were produced through sequestrations in surrogacy dormitories, where they were counselled and coached in order to inculcate an industrial discipline in them, which was central to accumulating surplus value.

Shifting her attention to how gestation and childbirth got incorporated into the market in India, Sharmila Rudrappa^4^ noted that the various actors involved were unsure if surrogacy was wage labour, or a gift relationship. Though many intended parents and surrogacy businesses posited that surrogacy in India was a gift relationship, market rules shaped the surrogacy experience. Relationships between clients and surrogate mothers were atomised and attended to mainly by money; when the intended parents believed they had met their obligations to the surrogate mothers, they ended their relationship upon making payments to the surrogacy agency once they received the baby. In the US too surrogacy was found to be structured as both a gift and a commodity exchange.^9,10,34^ Labour effort was compensated yet obscured under the cultural prominence of the gift narrative.

Surrogacy emerges only through specific forms of in-vivo extractive processes to which the surrogate mothers are subject. That is, sperm/egg banks, infertility specialists, and recruitment agencies facilitate medico-technical and legal interventions on the surrogate mother at the bodily, intimate, and emotional level so that their biological processes of oogenesis and gestation are managed for the clients’ benefit. Cooper and Waldby’s notion of “clinical labour”^1^ comes closest to capturing the labour effort entailed in surrogacy. Clinical labour describes the transformation of biological processes into abstract, interchangeable units complete with politically driven rates of exchange in the marketplace.^1^ Surrogate mothers, as clinical labourers, “offer themselves up as subjects, giving clinics access to the productivity of their in-vivo biology, the biological labour of living tissues and reproductive processes”.^2^

Yet how and why do women acquiesce to becoming clinical labourers? The agents involved in surrogacy operate within specific moral frames or imaginaries.^10,21,35,36^ These moral frames provide the ethical structures and reasoning within which firms, consumers, and surrogate mothers signify their actions.

### Moral frameworks

Surrogacy in our studies in the US, India, and Russia (as perhaps indeed other types of clinical labour too) came into existence, and into its actors’ lives, through locally specific normative cultural frameworks that varied across different regions. Rudrappa and Collins^12^ posited that surrogacy in India emerged from moral frameworks, which were schemes of interpretation that “enable actors to locate, perceive, identify, and label”^37^ events in their social worlds. Through such frameworks, individuals not only make sense of their worlds, but they also legitimise their actions to others, “thereby attempting to garner social sanction rather than disapproval”^12^ This mechanism was also foregrounded by Heather Jacobson^10^ in her ethnography of surrogacy in the US, where building friendships between surrogates and intended parents was perceived to be a socially palatable and therefore also ethical way of framing surrogacy.

The concept of moral frameworks helps us understand how actors use dominant normative imaginaries in their specific locally situated reproductive decision-making. In this respect, for instance, Elizabeth Roberts^38^ showed in her ethnography of in-vitro fertilisation (IVF) in Ecuador how the dominant Catholic religious morality was referred to in IVF clinics in conjunction with local economics of care, which in turn diminished the anxiety that could otherwise accompany a deployment of Catholic morality in the context of IVF.

These findings resonate with those feminist studies of reproduction which have long shown how debates about reproductive technologies are embedded within local moral worlds, and they are notably shaped by cultural, religious and other beliefs.^39,40^ For example, Susan Kahn^41^ showed how in Israel, users of reproductive technologies on one hand, and religious authorities on the other hand, re-negotiated the moral frameworks within which emerging reproductive technologies could be situated in pre-existing understandings of Jewish kinship.

Moral frameworks are also shaped by economics: feminist scholars of reproduction^42–44^ have shown how the gendered social division of labour in modernity has been linked to dominant views about women’s altruism and their availability for devalued reproductive labour. Within such a framework in the US, egg donors were found to be viewed by clinic professionals as more altruistic than sperm donors.^24^ In her analysis of “prochoice” and “prolife” activists in a Midwestern town in the US, Faye Ginsburg^45^ showed how moral views on abortion were linked to the structure of “productive” and “reproductive” labour in the society and to related meanings of activist labour: on both fronts, the activists created alternative life scripts to what they considered to be a conventional cultural form for a female trajectory.

Thus, we understand moral frameworks in multiple terms: as culturally dominant normative frames of reference and legitimisation that exist prior to the onset of a specific mode of labour such as surrogacy, but also as ideologies and meanings that are co-determined by the economic conditions they are part of.

### Repronational histories

The moral frameworks through which the clinical labour of surrogacy is negotiated within local gendered bio-economies^43^ could also be understood as a product of “repronational histories”:^20^ “a pattern of specific national events – such as the role of influential clinics, media coverage of IVF, public controversies and court cases, political and legal decisions, religious edicts, or maverick individuals/teams – moulding IVF provision into a specific shape”. Through collecting and analysing a series of national case studies of the emergence of IVF across the globe, Franklin and Inhorn showed how in each country IVF was developed through a different local set of societal influences, whilst at the same time reflecting the global power relations. They identified “the changing cultural values, norms and rationalities with which this technology is most closely linked”, as well as “how closely reproductive technologies are interwoven with projects of social, moral and national reproduction”.^20^ In the same vein, Laura Briggs^46^ and others have shown how reproductive politics underpin all politics, and how they are appropriated by nationalisms. This became particularly visible in the context of the right-wing politics of Trump and Brexit.^47^

### Transnational reproduction

Yet local moral frameworks and related gendered bio-economies are often either broader or narrower than a national jurisdiction. Charis Thompson^48^ showed how transnational and not only national circuits of technological innovation determine the ethics of stem-cell reproductive science. Global chains have been found to account for a multi-sited and often transnational nature of surrogacy and egg donation.^21,49,50^ In her research on surrogacy in the western Russian metropolis of St Petersburg, Christina Weis^7,51^ observed that the moral framework of worker-mothers referred to by the interviewed surrogate mothers dated back to the reproductive politics within the Soviet Union, which exceeded national jurisdictions.

### Repro-regional moral frameworks

On the other hand, studies of surrogacy found important regional differences within national jurisdictions. For example, as Anindita Majumdar found, kinship and gender structures in northern India could disempower surrogate mothers in ways that rarely existed in southern India.^4,22^ In the case of Russia, the only currently existing research is on surrogacy in the western cities of St Petersburg and Moscow, but surrogate mothers living in the metropolis were found to have different experiences to those who commuted to work as surrogate mothers,^7^ suggesting regional differences in how surrogacy is conceived within Russia. Such differences were also starkly visible through the different trajectories of surrogacy legislation in several US states, such as California and New York: whilst California had been building a global surrogacy industry and progressive legislation for almost three decades, it was only in 2020 that New York legalised commercial surrogacy.^52^

Also in line with these findings are our three studies from the western US state of California, the southern Indian state of Karnataka, and the western Russian metropolis of St Petersburg. We therefore argue that the moral frameworks used by our interviewees to interpret their involvement in surrogacy are “repro-regional”: they partly overlap with national jurisdictions and politics, partly exceed them, and partly refer only to the cultural or political region and bio-economy they are each set in. We suggest that these repro-regional moral frameworks overlap with cultural norms and histories that are dominant in the region, as cultural idioms that are invoked as interpretation schema and means of legitimisation. They can form a basis for social contracts around issues such as surrogacy, as suggested for example by Heather Jacobson^10^ in her analysis of the form of surrogacy that is socially acceptable in most of the US. We recognise that our understanding of “repro-regional moral frameworks” is still a broad category, yet we hope that alongside our comparative study, other future studies can show how repro-regional moral frameworks in specific locations can overlap with social hierarchies such as class, race, or other.

## Surrogacy contexts within the USA, India and Russia

Currently, commercial surrogacy is legal and available to intended parents of any gender, sexuality, civil status, and citizenship in several US states. Some states, notably California, have developed established surrogacy industries and laws over the last three decades, such as the pre-birth parental order that adjudicates parenting rights to intended parents as early as before the surrogacy baby’s birth. Importantly, several US states are also the only established destinations globally where gay intended parents can currently pursue surrogacy. Those states have therefore become destinations for both domestic and international reproductive travel.^53^ Nonetheless, the cost of gestational surrogacy in the US is $120,000–150,000, out of which the surrogate mother usually receives around $25,000–35,000.^34^

Though the US remains a critically important node, India has emerged as a strong contender on the global surrogacy circuit. Legalised in 2002 by administrative fiat, commercial surrogacy in India, with its estimated 3000 fertility clinics, was appraised as having garnered more than US $400 million in profits per year.^54^ India witnessed a burgeoning clientele over the years that led to its various monikers such as the world’s “baby destination”, “baby farm”, or “rent-a-womb” industry. In spite of operating on only regulations, and not legislative terms, India’s success in cornering the surrogacy market was not surprising; surrogacy cost between US $45,000 and $60,000 for a singleton baby, with surrogate mothers receiving anything between $4,000 and $10,000 depending on the region of the country. In 2012, however, surrogacy services started shrinking in India when gay couples and single parents were banned from receiving surrogacy assistance. By September 2016 this ban included all international clientele. At present altruistic surrogacy is available only to childless heterosexual Indian citizen couples. Commercial arrangements are banned in India.^55^

In Russia, clients are mostly citizens, but in response to the surrogacy bans in India, Thailand, Nepal and Mexico, Russian surrogacy agencies and private fertility clinics have increasingly been tailoring their surrogacy services for international clientele. However, although commercial surrogacy arrangements have been implemented in Russia since 1995, they remain poorly regulated.^7,51^ At first solely guided by medical guidelines, the first surrogacy-specific Federal Law No.323 was adopted in 2011. The law stipulates that only gestational surrogacy is legal, and the surrogate mother is the legal mother of the surrogacy-born child until she relinquishes the child to the intended parents by signing a specific notary-authorised form. With the signature and further documents provided by the IVF clinic that performed the embryo transfer and by the birth clinic where the surrogate mother delivered, the intended parents can establish their legal parenthood at a civil registry. At the time of data collection (2014/2015), a surrogacy arrangement in Russia cost US $35,000–60,000, of which the surrogate mother received between $10,000-17,000 after the delivery of a healthy baby, plus a monthly allowance of $250–400 for food and other expenses.

## Methods

Our methods of data collection varied since our three studies were conducted independently of each other. However, given our enquiries into global reproductive markets, we felt that it would be fruitful to collaborate in thinking through moral frameworks that shape the clinical labour involved in surrogacy in our three cases.

The researcher who worked in the US (Smietana) completed in-depth interviews with 20 surrogate mothers, 37 intended gay fathers who were carrying out surrogacy in the US (half living in the US, half in several countries in Western, Northern and Southern Europe), 15 assisted reproductive technology (ART) professionals, and four egg donors between 2014 and 2016, mainly in California. Interviewees were recruited through contacting surrogacy and ART clinics as well as associations formed by surrogates, intended parents and surrogacy families. The researcher worked more extensively with some interviewees, meeting them several times throughout their surrogacy journeys, and accompanying them to medical appointments or agency meetings. They also attended industry sponsored surrogacy workshops, clinic and agency anniversaries, and other surrogacy related events. All interviews were conducted in English.

The researcher in India (Rudrappa) conducted participant observation in an infertility clinic in Bangalore, South India, for two months in 2009; interviewed eight heterosexual and 12 gay individuals/couples from the US and Australia who travelled to Mumbai, Anand and Delhi in 2010–2012 for surrogacy purposes; conducted in-depth interviews with seven infertility specialists from Bangalore, Mumbai and Hyderabad and with three lawyers who facilitate surrogacy in India and in the US; and in 2011, interviewed 70 surrogate mothers, 31 egg donors, and 25 garment workers in Bangalore. Some of these interviews were more detailed because the researcher met with ten of the surrogate mothers numerous times. All interviews were conducted in English and Kannada.

The researcher in Russia (Weis) conducted 15 months of ethnographic fieldwork in St Petersburg, visiting fertility clinics, gynecological units, maternity wards and housing units for surrogate mothers between September 2012 and January 2013, and August 2014 and May 2015. The researcher interviewed 40 surrogate mothers; eight client parents; 11 infertility specialists; 14 members of staff of surrogacy agencies; three maternity clinic staff members, two lawyers and staff at the German consulate. The interviews with surrogate mothers were conducted in Russian and Romanian; and the interviews with client parents, agency and medical staff in Russian, English and German.

In the three studies, the qualitative interview and observational data were transcribed and analysed thematically. Following the ethnographic tradition of reproductive studies^4,45,56^, the main themes that emerged from the analysis were written up in the narrative form, where due attention was given both to specific cases and to their comparison to other cases within each study.

One common denominator across our three studies was that in each case we asked our interviewees how they understood the labour of surrogacy and what it meant to them. In the subsequent writing up process of this comparative article, we stuck to the thick descriptions coming from each of our three individual projects, whilst we aimed to contrast the meanings and ideologies through which the clinical labour of surrogacy was understood in each of the three contexts we analysed. We decided not to pull apart the narratives of each of our three ethnographies into a more schematised comparative grid or structure, as such a structure could risk depriving each of our repro-regional stories of its specificity, given each ethnography was embedded in its context in a unique way. Following the ethnographic tradition of thick description^57^, we present below the three cases we researched: the US state of California, the southern Indian state of Karnataka, and the Western Russian metropolis of St Petersburg.

## Surrogacy in the US: angels make a living

In celebration of their anniversary, Agency X organised a Sunday picnic for surrogates, intended parents and other collaborators who intermingled in an environment that was leisurely and family-like. The picnic area was decorated with a line of hand-made posters with the photos of the surrogates and families the agency helped create over the years, and games and balloons for children who played. Several adults chatted at picnic tables in the spacious green park. In the blaze of the summer sun, the agency founder and owner, Jodie,† herself a surrogate three decades earlier, hushed the crowd as she announced in an emotional voice, “Thanks to all of you. And first of all, to our surrogates, for your sacrifice and the gift of life you have given to help create families. You are angels!” Jodie’s metaphor of angels recurred repeatedly in the narrative of altruistic gift giving in various events organised by surrogacy agencies, infertility clinics and other associated events, as well as from the 20 American surrogates interviewed over an 18-month period between 2014 and 2016.

“I’m a surrogate because I love to help people, and I’m proud to be a surrogate”, said Linda over a restaurant dinner in a small town in southern California. She said that it was her desire to help others achieve what she had; she was the married mother of two teenagers and could not imagine life without them. Her very first surrogacy agreement surprisingly did not go very well; right after childbirth the intended parents cut off all ties with her. She said that she “couldn’t leave on a note of a couple treating me like I was their employee … they got what they wanted and I’m fired”. This motivated Linda to continue, and in her subsequent arrangements she was successful in forging satisfactory relationships with the families she helped. Whether relationships were closer or a bit more distant, they are always “kind of like a family”, she said. With a stable job in local administration, Linda recently turned forty. After five gestational surrogacy arrangements, she was retiring from surrogacy.

Indeed, although surrogacy in the US was achieved through labour contract, much like Linda’s case, almost all surrogates sought an alignment of values with clients in order to form kin-like relationships. Gay fathers Mike and Bob, and their second-time surrogate, Kath, were illustrative. The men wanted somebody “who seemed like they’re safe, and they’re sane, and healthy. And we really consider ourselves close friends, but she’s not gonna do this as a charity, she needs to provide for her family too”. Mike recalled their first meeting with Kath:

“We had coffee in Starbucks about a mile from her house after work, and we talked about it … it was a very efficient conversation, like what do you care about, and we talked about religion, we talked about food, we talked about fetal reductions that we didn’t want unless there was a danger to her health, we talked about, you know, like illness and things that she would control in the decision-making process, related to the health of the baby and the surrogate, what would we do in different scenarios – and I think once we aligned on those big things, then we were like ‘ok, let’s contract’.”

Depositing $50,000 in an escrow account for Kath, which included expenses for pumping breast milk, organic food, and other incidentals, did not reduce their relationship to pure instrumentality. Kath explained her motivations for being a surrogate mother, ranging from having a gay brother herself to her passion for home schooling and planning to foster a child in the future. Mike and Bob echoed Kath, insisting that what made them decide to work with Kath was “the gut feeling”, “a trust move”, and an alignment of values. Two years after the birth of a baby boy for the gay couple, the two families visited each other twice a year, exchanged pictures, and chatted on Facebook. Mike and Bob sent Kath’s family Christmas presents.

Relationships such as that between the couple and Kath were initiated with online profiles much like online dating. Kate, a full-time schoolteacher and wife to a small entrepreneur living in a small northwestern coastal town exemplified this scenario. Her online profile read in the following manner:

“Hello! My name is Kate and I am a 35 year-old mother of three. Most people would describe me as nice, loving, caring, and funny! … As far as what I’m looking for in a couple is honesty and trust … I feel like we can accomplish anything working together as a team!”

Intended parents also posted their profiles with the objective of “finding someone you can just get on well with, and have a similar approach to pregnancy and mutual relationships,” Kate said.

Even if some women did not stay in regular contact with their surrogacy “kin”, they exchanged Christmas cards, were Facebook friends, kept photo albums attesting to the “kin connection”, and made holiday phone calls or even visits. Through these narratives of gift-giving and seemingly mutually respectful relationships, commercial surrogacy contracts were affectively de-commodified and deemed socially acceptable.^34^ Even if approximately US $30,000 were exchanged, surrogacy was never framed simply as labour. Rather, it was a “labour of love”.^10^

What is more, describing surrogacy only through the lens of wage work and money was met with disapproval by industry stalwarts in the US. Susie, a well-known surrogacy attorney and counsellor noted that “if in the initial screening interview with surrogate candidates I sense that their only motivation is money, I reject them. It won’t work”. Susie was not alone; time and again various participants reiterated that they engaged in surrogacy not just for money, but for emotional, and spiritual rewards. Agencies and clinics were also careful not to recruit women without stable family income, so as to avoid economic pressure on them.

Yet, three of the 20 surrogates interviewed in the US struggled with medical insurance claims that their surrogacy agencies had not adequately sorted out. Their surrogacy contracts authorised them to seek compensation from the intended parents, but that was not easy given the transnational nature of the agreements. Tina, just after the birth of her surrogated child, was scheduling various job interviews. She worried about her job prospects because her earning from surrogacy was running out as she struggled to make ends meet for herself and her young child.

The “angels” had to make a living. Linda, the five-time surrogate, acknowledged that her first foray into surrogacy was initiated by financial concerns. “After seeing an ad for surrogacy in the Penny Saver” she noted, she turned to her husband and said, “Here’s a way I can make some money, and he said, how? and I said, I could be a surrogate, so he says, Well, why don’t you? and I said, Fine, I will!” Negotiation processes between spouses are not always as straightforward, but all 20 American women interviewed highlighted the importance of the economic compensation.‡ “I’ve been compensated for my time and energy throughout the treatment with monthly payments”, explained Linda. Like most others interviewed, Linda had spent her surrogacy wages on a down payment for a home, and home improvements. On the other hand, northern Californian Kate invested her earnings in a family business. All 20 surrogates said that their earnings contributed to everyday family expenses.

Given that surrogacy could in many cases be considered as a feminised part-time job, which provided a much-needed injection of cash to the surrogates’ lower or precarious middle-class family budgets,^34^ it was not surprising that both surrogates and intended parents in the US interchangeably used the words “help” and “work” when speaking about their surrogacy arrangements. For them it was labour, but one which was special and required an emotional investment. The affective narrative of gift giving co-existed with the market narratives of compensation. Much like other studies on American surrogacy^9,10,35^, the fieldwork in the US indicated that for surrogacy to be socially acceptable, it had to be framed as a de-commodified exchange mediated through altruism and relatedness. Such a reproductive imaginary resolved the tension around the cultural anxiety about the commodification of life latent in the surrogate’s alienation from the product of her labour, the child. As much as a moral framework of this kind has been used by some of the interviewees to forge meaningful relationships, by some others it has been used only as a narrative tool for understanding and legitimising surrogacy or, at most, giving it a personal touch. Such diverse uses of surrogacy in California were possible in the context of broader reproductive politics in the US, which, as other scholars have shown, has been framed by narratives of individualism and liberalism^38,46,58^ and indeed perhaps even a morality of individualism and liberalism.^59,60^

At the same time, such an imaginary may have obscured the labour involved.^10^ As Cooper and Waldby ^1^ point out, “the ethical insistence that *the biological should not be waged*” may be responsible for “atavistic … forms of labour contract and desultory forms of compensation”. American surrogates’ locations in the gendered bio-economy were precarious and flexible, and they were post-Fordist workers par excellence: they signed fixed-term contracts, and continual wages were conditional upon the achievement of performance, that is, delivery of a baby.^1^

## Surrogacy as “philanthropy”: the case of India

Amidst criticism that she exploited indigent women, Dr Nayna Patel, the doyen of Indian surrogacy, echoed a common trope in circulation among infertility doctors, surrogacy agencies, intended parents, and the media:

“There is nothing immoral or wrong … A woman is helping another woman, one who does not have the capacity to have a baby and the other who lacks the capacity to lead a good life … when the end result is a lovely baby how can you say there is something wrong happening … [surrogate mothers are] able to buy a house, educate their children and even start a small business … . It’s a win-win situation.”^53^.

Married gay couple Phil and Colin, who lived in New York with their three six-year old children born through two surrogate mothers in Delhi, said they weighed the moral implications of going to India. They argued against domestic surrogacy because although a US surrogate might receive around $25,000, it did not change her life as it did not amount to a large percentage of her overall family income. In India, though, Colin guessed that the women received $4000–5000 for being a surrogate mother, which was “ … the equivalent of maybe five years of income. It enabled them to move into a home, to get an education for their children”. He rhetorically asked those who questioned his decision to go to India: “What have you ever done to make the lives of these women better? You are so quick to judge me, but I have. I can point to two people who have homes and have sent their kids to school as a result of our direct involvement with them”. Colin concluded that people who are “unfamiliar with the extreme poverty in India” did not realise that “there are a whole lot of winners here. No one was hurt”.

While medical providers and consumers justified their engagement in surrogacy in this manner, Rudrappa^4^ notes that the surrogate mothers she interviewed in Gujarat perceived their work as “god’s labour”, and incomparable “gifts for global sisters”. Surrogate mothers interviewed in Bangalore, however, posited surrogacy as a personal moral choice. When asked by the researcher why she wanted to become a surrogate mother, the 25-year-old garment worker Ramaa explained that she needed money for better housing, to pay for private schooling for her two children, and to repay usurious money lenders to whom she and her husband owed money. But there was another reason. She said that one afternoon during Bangalore’s monsoons her family was disturbed by raucous stray dogs fighting by the pile of trash at her street corner. Puzzled by why the dogs were attacking each other in torrential rain rather than seeking shelter as they are wont to do, Ramaa’s husband went out to investigate. He shooed away the dogs, and found what they were battling over; in the pile of trash, spilling out of a torn plastic bag were the remains of a foetus. Then, looking contemptuously at the researcher who had dared to ask her to explain her decision, but was now shocked into silence, Ramaa concluded, “Now you know why I am a surrogate mother. I value life more than most people around me. I create life”.

Many interviewees in Bangalore too marked the inherent morality involved in surrogacy, but not in ways as dramatic as Ramaa. Instead, they located their work as surrogate mothers in the context of their personal labour histories as garment workers. A large number of surrogate mothers in Bangalore had been recruited from sweatshops that produced garments for global retailers, where women were treated as disposable workers.^61^ In order to meet short production cycles set by global market demands, the women worked at an inhumanely fast pace, and with few breaks. Like elsewhere, garment workers in Bangalore too were underpaid and overworked. Male supervisors used sexual harassment as a technique to instil shop floor discipline. Indirani, a 30-year-old garment worker and surrogate mother, exemplified what many Bangalore surrogate mothers said. Married at 18, and with two young children of her own, Indirani and her husband struggled to make ends meet. The couple borrowed money from Indirani’s cousin, but their troubles worsened because they were unable to pay back the loan. Indirani first provided her ova for US $500, and then signed on as a surrogate mother. At the first attempt she got pregnant and birthed twins for an Indian couple who lived in the US.

Indirani did not find surrogacy to be debasing work. Because she stayed in the surrogacy dormitory, she was far less physically and emotionally exhausted than as a garment worker when she had to return home to the “second shift” that involved taking care of her family. As a surrogate mother she stayed in a dormitory with others like her and had no household obligations. Her mother took care of her children, while in the dormitory she had the luxury of being served by others. Indirani contrasted garment work to producing a baby. “Garments?” she asked rhetorically, “you wear your shirt a few months and you throw it away. But I make you a baby? You keep that for life. I have made something so much bigger than anything I could ever make in the factory”. Indirani observed that the people who bought the garments she made probably never thought of her. On the other hand, she was etched forever in the minds of the intended parents for whom she had birthed twins. She had changed their lives because she had fulfilled a woman’s desire to be a mother, secured that woman’s marriage, and guaranteed the continuity of a patriarchal bloodline.

Indirani and the other surrogate mothers did not misread their exploitation. They were aware of how their surrogacy work was being disregarded in the context of labour rights, and some of them used the word “exploitation” themselves. However, given their meagre employment options, they believed that surrogacy afforded them greater control over their emotional, financial and sexual lives. Surrogacy was a profoundly creative labour that allowed women to assert their moral worth. At the dormitory, they were in a women-only space, in contrast to garment sweatshops where sexual harassment was extensive. Moreover, they made babies without engaging in sex, which added to their appreciation of the immense creative capacities they held in their bodies. As a garment worker Indirani said she was being destroyed, but as a surrogate mother she felt had created a new world. She wanted to be a surrogate mother again, this time to earn money for her children’s private schooling. Indirani felt in control.

Yet, this fervently held belief that they had greater control over the labour process in surrogacy did not pan out when birth outcomes ended tragically. Sita, a 26-year-old homemaker, was a surrogate mother because she was in urgent need of cash after a car accident that resulted in her six-month-old daughter’s death, and left her husband with life-threatening head injuries. At her last meeting with the interviewer Sita did not look well. She had birthed two babies through caesarean surgery ten days earlier, and she had just learned that one of the twin babies had died. She had repeatedly told the doctors that she could not feel one of the babies move, but they did not believe her. She now mourned furiously that if the doctors had paid attention and performed her caesarean surgery earlier, as she had requested, the outcome for the infants would have been better. She expressed that all the work she had put in birthing the babies had amounted to nothing. The two fathers who had contracted with her had never met with her throughout her pregnancy. She was forbidden from visiting the surrogacy baby she had birthed. She expressed that she had lost two babies: her six-month old daughter in the car accident, and this premature baby boy at birth. Unlike Indirani, who had birthed twins successfully and felt in greater control, Sita and the twins she birthed faced heartbreaking outcomes, which left her feeling powerless. Thus, surrogate mothers in India felt surrogacy afforded them greater choice and control in how to use their reproductive capacities. Yet, the industry was structured in such a way as to minimise surrogate mothers’ control over the labour process. This included control over their relationships with intended parents, both in those cases where surrogate mothers desired such relationships and in cases where they did not express willingness to establish any such ties with intended parents.

By and large, though, in spite of the way the industry was organised, the surrogate mothers in Bangalore made sense of their clinical labour through the narrative of giving life, as opposed to the life-devaluing context and conditions of the global garment industry in which many of them had been working. The women understood the clinical labour, the in-vivo work they performed, as an immensely creative and life-affirming labour process.

## Surrogacy as a business arrangement in Russia

**“**This is my work”, Anna answered, a little surprised and unreservedly, when faced with the researcher’s question of what surrogacy meant for her. For Anna, a married stay-at-home mother of two, and five months pregnant with twins in her second surrogacy arrangement, it was self-evident that gestating someone else’s child for financial compensation was a temporary employment. Upon the researcher’s further probing “When someone discovers for the first time that you are a *surmama*, how do you explain what you are doing?” Anna reiterated, “That I work. For me, this is work. I don’t see anything else in it”. Managing the preparatory hormones, conceiving, monitoring the pregnancy, complying with various appointments and medical and dietary recommendations and, finally, giving birth, was for Anna and many of the surrogates in St Petersburg just another job. Anna planned on using the surrogacy-earned money to buy an apartment because she was tired of cramming her family of four into the three-room apartment she shared with her sister’s family of three. She compared her home to a “*kommunalka*”, the Soviet communal living apartment, notorious for crowded conditions and lack of privacy. Likewise, single mother Daria stated, “I do [surrogacy] for the money. It’s a job of certain sorts”. Unlike American or Indian surrogate mothers, then, Russian women perceived surrogacy as a job, which at some level, was how motherhood in general was perceived.

In Soviet Russia, women’s role was defined as worker-mothers, whose duty was to work and to produce future generations of workers. In return for their service of motherhood to the state, mothers received money and state-subsidised services.^62^ This historical framing of motherhood as work in Soviet Russia may have paved the way for established cultural narratives through which worker and mother identities could be seen as not necessarily dichotomous. In post-Soviet Russia, an immediate consequence of the new system was the depreciation of motherhood. There were massive cuts to subsidised childcare, pushing working mothers to drop out of employment. Many women had to find employment that helped them balance motherhood with paid work, and they sought alternative, mostly family-supported childcare arrangements.^63^ Within this context, surrogacy workers in Russia approached surrogacy as an opportunity to earn money in their capacity as mothers. In this process, they could refer to the already existent local moral frameworks which legitimised their identities of mothers as workers. Like many of the *surmamas* interviewed, Anna and Ilya did not consider making a living by creating life morally questionable. The average payments for successful pregnancies of US $10,000–17,000 exceeded their income possibilities through regular employment in the same time period.

Women’s financial motivations were a crucial selection criterion for commercial agencies. Grigory, owner of agency A, stated:

“[Surrogacy] is a paid service. And of course it’s work (…) it’s a job - one of the most responsible jobs in the world and of course the surrogate should get the right remuneration. (…) [The ideal surrogate mother desires] to help childless people to become parents. And, of course, she should not be altruistic. She should wish to get money for herself, for her family, for her own children. And if she enjoys being pregnant (…) that makes her an ideal surrogate.”

Surrogate recruits were expected to have a business attitude, as altruism and the notion of surrogacy as gift invoke expectations of reciprocity and a relationship. In order to prevent arrangements from getting messy, agencies advised client parents to avoid developing friendships with their surrogacy worker. Surrogacy workers who expressed a preference to remain strangers with their client parents were preferred by recruitment agencies. Agency staff maintained that financial motivation made women better surrogates because they carefully adhered to instructions in order to maximise their wages. Alexander, owner of agency B, explained that surrogacy contracts stipulated monetary fines for transgressions such as substance abuse, or neglecting a prescribed diet. Upon the first violation, a surrogacy worker lost a minimum 10% of her prospective earnings. “Our Russian girls only speak Ruble”, said Alexander’s co-worker Elena as a joking expression of her belief that financial threats worked with *surmamas*.

The women were hyper-aware of the workload involved in surrogacy. Surrogacy workers in Russia compared their gestational labour with child-minding to highlight the work character, and the caring nature of their work. “I was like a nanny, a good nanny at the time of the pregnancy. But those twins were entirely their children”, said Daria, and emphasised that nannies often spend more time with children than the parents, without claiming the children to be theirs.

Oksana, who also drew on the nanny comparison, explained the challenging and precarious nature of surrogacy work.

“It was a difficult pregnancy. I felt bad, slept a lot, had to take the pills, all those hormones - and it is not your pregnancy. You worry a lot! All the time you are tense, you are worried that nothing will happen, that - God forbid … [Silence.] You carry such a responsibility.”

Aware of the workload, the precariousness and the emotional labour, surrogacy workers in Russia bargained for a satisfactory compensation, and called out client parents or clinics who offered as little as US $7000 for their gestational services. Daria’s remark about an agency offer that she refused illustrates:

“One thing surprises me: the underrated pay they offer. 600,000–700,000 Rubles. But guys, you need to understand that 3 years ago, 600,000 was US $20,000. Today, US $20,000 is about a 1,000,000 Rubles. What are we talking here about!? And when I come as an experienced surmama and guarantee a good health (…) and I am told that 1,000,000 is a lot?! I am not ready to enter a program for that little. Then I’d better not go at all!”

Perceiving surrogacy pregnancy as a form of work, rather than as an altruistic act, was reflected in the relationship between client parents and surrogacy workers. Anna described her interactions with Svetlana, the first client she had worked with. Unlike in Anna’s second arrangement, during which the client parents maintained strictest anonymity and paid the agency to fully assume the tasks of monitoring her, her first client, Svetlana, had chosen a so-called minimum package. In such arrangements, the agency only selects a surrogacy worker and arranges the embryo transfer. Once pregnant, the client takes charge of the course of the pregnancy, arranges appointments, birth preparations and the administrative duties of registering their child. Anna elaborated:

“[During the pregnancy] we didn’t just call each other to chat about needless stuff. But when I needed something – I called. When she wanted something – she called. That means, on business. As they say, we talked business.”

Surrogacy worker Lyubov referred to her client parents as decent people who were “not of a chatty nature”. Their communication was neutral and “business-like”. When her client mother called, usually in regular weekly intervals or to follow up on pregnancy examinations that she missed, she avoided emotional matters and focused on quantifiable information such as Lyubov’s weight gain and the girth of her protruding belly. Lyubov commented, “I have enough friends [to find support when I need it]. Surrogacy – that is work. (…) Communication limited to business has its advantages: it won’t be so difficult to part later”.

The notion of surrogacy as an economic exchange ^64^ also shaped Russian client parents’ and surrogacy workers’ expectations after childbirth. Upon childbirth, client mother Svetlana paid one last visit to Anna in the clinic when she came to pick up her baby and Anna’s written relinquishment of her parental rights. Svetlana paid Anna, and thereafter, contact between the two women ceased. Anna destroyed the surrogacy contract because it had Svetlana’s contact details. She stated, “If she wants to stay in touch, it should happen on her initiative”. This attitude was widely shared. For instance, Ilya was taken aback when asked if she intended to maintain contact with her third client parents, with whom she had a cordial relationship. She said:

“I have never taken the initiative! That would be disreputable, or, at a minimum, not nice. (…) [The parents] will express words of gratitude, and express their gratitude in material gifts … and then they will return to their lives (…). In essence: what place do I have in it?”

Anyuta described her role in the relationship as “[being] like a hired worker”. Announcing, “We will part forever!” she said she intended to sever ties with her clients immediately after delivery, ideally before the client parents themselves would. The relationship had to be transient. Thus, the clinical labour entailed in Russian surrogacy was framed through the moral lens of a non-altruistic business-like commodity exchange.

## Conclusions

In this paper, we provided ethnographic interview data from the three studies of surrogacy we had carried out separately in the western US state of California, the south Indian state of Karnataka, and the western Russian metropolis of St Petersburg. Within each location, we were able to distinguish an overarching ethical approach to surrogacy, which we subsequently called a “repro-regional moral framework”, building on the literature on “moral frameworks” ^12^ and “repronational histories”, ^20^ yet doing justice to the regionally situated character of the moral frameworks of surrogacy beyond the national context only. The repro-regional moral frameworks we identified in our interviewees’ narratives each constituted an operational ethical framework of surrogacy business in the region, which took into account local societal values.

Surrogacy in our studies in the US, India, and Russia (as perhaps other types of clinical labour too) came into existence, and into its actors’ lives, through locally specific normative cultural frameworks that varied across different regions. The surrogate mothers in our studies – across the world in the US, India, and Russia – experienced the need to have better housing, provide better opportunities for themselves and their children, and have better lives overall than what they could access. But what compelled them were notions of what this work meant to them, and who they were as individuals that provided gestational services for a fee. We began this article with the question of how surrogate mothers, intended parents and surrogacy professionals understood their participation in surrogacy labour markets, and we provide a partial answer by analysing the moral frameworks used by American surrogates in California as a “labour of love”; by Indian surrogate mothers in Karnataka as an immensely moral and life-affirming act; or, simply as a job by Russian *surmamas* in St Petersburg. These, we propose, illustrate the specific repro-regional moral frameworks within which surrogacy emerges as a form of clinical labour.

Our interviewees’ ethical reasoning about their participation in surrogacy was in each case set in the context of gendered bio-economies^43^ of clinical labour^1^, which was precarious post-Fordist work per excellence. The surrogate mothers we interviewed regarded surrogacy as labour in all three contexts, while their views on other dimensions of surrogacy varied depending on the regional context: e.g. friendship with intended parents was also part of the surrogacy moral framework in California, whilst Indian surrogate mothers in Karnataka considered their work to be not only labour but also a life-affirming act.

Our comparative analysis of the narratives among surrogacy participants in California, Karnataka and St Petersburg provides a critical intervention in studies on global surrogacy, where time limitations, funding, language limitations and a plethora of other real barriers exist to conducting global comparisons. Such comparative perspectives, we believe, not only further an understanding of how global surrogacy works, but also, by throwing into comparative relief, illuminate the particular idiosyncrasies and specificities of the cases of California in the US, Karnataka in India, and St Petersburg in Russia. By introducing the concept of repro-regional moral frameworks we wish to emphasise the cultural, historical, and political specificities of how surrogacy emerges within given contexts. We acknowledge, though, that thicker ethnographic details than what we could provide here are fundamental to furthering an understanding of repro-regional moral frameworks.

Building on these findings, we argue that any international or global regulation of surrogacy, or indeed any moral stance on it, needs to take the repro-regional moral frameworks into account. For example, if there existed any kind of a global convention on surrogacy, in line with the existent global regulation of inter-country adoption,^17^ it should not stipulate that surrogate mothers and intended parents necessarily form friendship relationships as in Californian surrogacy. It is probable that importing such gift-like commodity relationships to St Petersburg would not work, given the embeddedness of the business-like commodity exchange model in western Russian surrogacy.

Our work points to the immense difficulty of coming up with an international policy framework for surrogacy, such as “Fair Trade International Surrogacy”^18^ or the proposals from the International Forum on Intercountry Adoption and Global Surrogacy.^17^ We suggest that it may not be possible to come up with a global convention on surrogacy and thus, unfortunately, in the case of surrogacy (unlike adoption), much policy needs to be regional, or at the most national. One key difficulty is that we cannot say whose moral framework is going to take precedence in a potential global policy; indeed, having any kind of policy intervention at all could be construed as ethnocentric. In addition, given surrogacy’s embeddedness in local economies and histories, interventions are needed into how business is conducted generally, and not only with regard to the surrogacy industry.

As ethnographers, we have provided and compared data from three different locations. We hope these data can be used by policy-makers to safeguard the sexual and reproductive health and rights of surrogate mothers, egg donors and intended parents involved in surrogacy in different locations across the globe, alongside the rights of surrogacy-born children. Our work points to the importance of local feminist struggles and bioethical interventions at the regional and national level to achieve a just form of surrogacy.

## Footnotes

* In the introductory and final sections of this paper, we use common English-language terms “surrogate mothers” and “intended parents”. However, in the specific sections on the surrogacy contexts in the three different locations we follow the terms used by our interviewees in each context, e.g. “surrogates” in the US, “surrogate mothers” in India, and “*surmamas*” in Russia; as well as “intended parents” in the US and India and “client parents” in Russia.

† All interviewees’ names are pseudonyms.

‡ This and the other two studies in this article did not include interviews with participants in altruistic surrogacy arrangements, which, however, do exist not only in jurisdictions such as the UK that have legalised only altruistic surrogacy models, but also within those jurisdictions where commercial surrogacy is legal such as many of the US states, Russia, and India at the time of this research.

## Research Data

For any queries regarding access to data used in this article please contact the corresponding author Marcin Smietana at ms935@cam.ac.uk or marcin.w.smietana@gmail.com
